# Evolution of sex in crops: recurrent scrap and rebuild

**DOI:** 10.1270/jsbbs.22082

**Published:** 2023-05-09

**Authors:** Kanae Masuda, Takashi Akagi

**Affiliations:** 1 Graduate School of Environmental and Life Science, Okayama University, Okayama 700-8530, Japan; 2 JST, PRESTO, Kawaguchi, Saitama 332-0012, Japan

**Keywords:** sex determination, hermaphrodite, dioecy, evolutionary dead-end, polyploidization, domestication

## Abstract

Sexuality is the main strategy for maintaining genetic diversity within a species. In flowering plants (angiosperms), sexuality is derived from ancestral hermaphroditism and multiple sexualities can be expressed in an individual. The mechanisms conferring chromosomal sex determination in plants (or dioecy) have been studied for over a century by both biologists and agricultural scientists, given the importance of this field for crop cultivation and breeding. Despite extensive research, the sex determining gene(s) in plants had not been identified until recently. In this review, we dissect plant sex evolution and determining systems, with a focus on crop species. We introduced classic studies with theoretical, genetic, and cytogenic approaches, as well as more recent research using advanced molecular and genomic techniques. Plants have undergone very frequent transitions into, and out of, dioecy. Although only a few sex determinants have been identified in plants, an integrative viewpoint on their evolutionary trends suggests that recurrent neofunctionalization events are potentially common, in a “scrap and (re)build” cycle. We also discuss the potential association between crop domestication and transitions in sexual systems. We focus on the contribution of duplication events, which are particularly frequent in plant taxa, as a trigger for the creation of new sexual systems.

## History, theoretical frameworks, and questions regarding sex evolution in angiosperms

### Transitions into and out of dioecy

Sexual systems in plants are clearly more diverse and complicated than in animals. In animals, an individual basically has only one sexuality, while individual flowering plants can exhibit multiple sexualities. This diversity allows for the potential coexistence of male, female, and hermaphroditic flowers (perfect flowers with both sexes in a flower). Thus, in plants, sexual systems in a species include a wide range of categories, such as hermaphroditism (only hermaphroditic individuals in a species) and many classes of separate sexualities, represented by dioecious (male and female individuals), monoecious (male and female flowers in an individual), gynodioecious (individuals carrying only hermaphroditic flowers or only female flowers), androdioecious (individuals carrying only hermaphroditic flowers or only male flowers). Hermaphroditism accounts for >75% of angiosperms and is thought to be the ancestral state of sexuality (Ming *et al.* 2011). Various outcrossing mechanisms promote genetic diversity within a species, as a matter of nature in plant evolution (Darwin 1877a). Temporal separation of the maturation of male or female organs, self-incompatibility, flower structural avoidance, monoecy, and ultimately dioecy, have all evolved from ancestral functional hermaphroditism (Ming *et al.* 2007). Dioecy accounts for only approximately 6% of angiosperms but is distributed across almost half (43%) of all angiosperm families (Charlesworth 2002, Renner 2014), which can be defined as convergent evolution. Dioecy has often been viewed as an endpoint of sexual system evolution (Heilbuth 2000). The relatively limited occurrence of dioecy in angiosperms is considered to be a consequence of two main disadvantages: (i) the lack of reproductive assurance of individuals, and (ii) only half of the population (female plants) produce offspring (Käfer *et al.* 2014). These considerations led to the hypothesis that transition to dioecy would be “an evolutionary dead-end”, with higher probabilities of extinction and lower diversification (Heilbuth 2000). However, some theoretical and literal studies have suggested that transitions out of dioecy may also be possible (Barrett 2013, Goldberg *et al.* 2017, Käfer *et al.* 2017, Pannell and Jordan 2022). Such a process could be represented as conversion from dioecy into gynodioecy, androdioecy, monoecy, or hermaphroditism, especially under domestication (e.g., papaya or grape, as discussed in more detail later). Lloyd (1975) first documented reversions from dioecy to monoecy in three species of the genus *Cotula*, which has been followed by several examples in other lineages. In the genus *Momordica*, phylogenetic analysis of the evolutionary history of its sexual systems suggested seven independent transitions from dioecy to monoecy (Schaefer and Renner 2010). In *Mercurialis annua*, diploid populations are basically dioecious, whereas androdioecy and monoecy are derived in polyploid populations (Russell and Pannell 2015). Despite these recurrent transitions into and out of dioecy in angiosperms, little is known about the molecular mechanisms that trigger them, including the establishment of sex-determining genes.

### Sex chromosome evolution

The establishment of dioecy in plants has attracted the attention of biologists for over a century. In 1903, a genetic sex determination system, suggested to be chromosomal sex determination as seen in animals, was first identified in *Bryonia dioica* (family *Cucurbitaceae*) (Correns 1903). Sex chromosomes in angiosperms were simultaneously discovered in 1923 in three different genera: *Silene*, *Rumex*, and *Humulus* (reviewed by Westergaard (1958)). White campion (*Silene latifolia*) has heteromorphic sex chromosomes with a heterogametic male system (XY system) (Blackburn 1923, Winge 1923). Sorrel (*Rumex acetosa*) and hops (*Humulus lupulus*) have an X:A dosage system, in which an X-to-autosome ratio of 1.0 or higher results in a female, and a ratio of 0.5 or lower results in a male (Kihara and Ono 1923, Winge 1923). Sex chromosomes have been identified, mainly from a viewpoint of cytogenesis, in over 50 species to date (Ming *et al.* 2011, Muyle *et al.* 2017, Renner 2014). Conventional observations suggested that degradation and heteromorphism of sex chromosomes could potentially result from strong sexual selection to establish (or maintain) linkages to sexually antagonistic (SA) mutations (Barrett and Hough 2013). In other words, establishment of sexual dimorphism might be a driving force for the formation of sex chromosomes. For instance, only 10 million years could have facilitated the very long male-specific region of the Y-chromosome in *Silene latifolia* (Ming *et al.* 2011). This tendency may not be generally applicable to any chromosomal sex determination, however, as suggested in recent independent hypotheses for the formation of (plant) sex chromosomes (Charlesworth 2019, Lenormand and Roze 2022, Renner and Müller 2021). Among the dioecious species, heterogametic males (XY) are thought to be predominant (84.7%), while female heterogamety (ZW) only comprises 15.3% of species (Leite Montalvão *et al.* 2021). Importantly, frequent turnover in heterogametic systems has been reported in several lineages. The genera *Dioscorea*, *Populus*, *Salix*, and *Silene* all include species with XY and ZW systems (Leite Montalvão *et al.* 2021). A theoretical model suggested that transitions between heterogametic systems may be selected when sex chromosomes are degenerated and the heterozygous sex has low fitness (Blaser *et al.* 2014). Another possibility is that when sexually antagonistic polymorphism is maintained on an autosome, a new sex determinant that arises in the region becomes advantageous (Van Doorn and Kirkpatrick 2007). Although genome sequencing of sex chromosomes was a difficult task until five years ago, recent developments in high-throughput sequencing technology have allowed (almost) perfect construction of sex chromosomes in some species, as discussed later.

### How did plants evolve sex?

One of the most representative theoretical models, the “two-mutation” model (Charlesworth and Charlesworth 1978a, Westergaard 1958), proposes the evolution of dioecy via gynodioecy. In this model, dioecy is thought to be established via a male-sterility mutation (often called disruption of the “M factor”) in an ancestral, functionally hermaphroditic state and, thereafter, a gain-of-function mutation for suppressing feminization (often called “SuF”) (Charlesworth and Charlesworth 1978a). Although this “two-mutation” model is predominant in discussions of plant sex evolution, the same authors also proposed another theoretical model for the evolution of dioecy with a single factor, via monoecy–paradioecy (Charlesworth 2013, Charlesworth and Charlesworth 1978b). Indeed, dioecy has been artificially engineered in monoecious maize (*Zea mays*) (Jones 1934) and cucurbit (Boualem *et al.* 2015). In any models, resource allocation and the avoidance of inbreeding are presumably both involved whenever a dioecious species evolves from a hermaphroditic species (Charlesworth and Guttman 1999). To verify these models, understanding of the evolution of sex determining gene(s) located on sex chromosomes would be indispensable. However, the sex determinants in plants have been identified in relatively few species, and evolutionary paths are still poorly defined (Renner and Müller 2021). Understanding the mechanisms behind sex determination is important not only for improving pollination success or controlling yield and fruit quality (fruit shape, or parthenocarpy), but also for planning cropping or breeding strategies. The detailed mechanisms for different sex determining gene(s) in crops are discussed in the next section.

Another important question concerns the effect of polyploidy on sexual systems (or transitions into and out of dioecy) (Ashman *et al.* 2013, Glick *et al.* 2016). The association between dioecy and polyploidy was documented by Baker (1984) and was highlighted by Miller and Venable (2000). Polyploidy is frequently found in several plant clades in which sex chromosomes have been well studied [e.g., in the genera *Salix* (Wagner *et al.* 2020), *Silene* (Popp and Oxelman 2007), *Mercurialis* (Russell and Pannell 2015), *Diospyros* (Akagi *et al.* 2016a), and *Fragaria* (Tennessen *et al.* 2018)]. Polyploidy can affect genome evolution and gene expression, such as chromosomal rearrangements and gene loss (Udall and Wendel 2006), unequal rates of sequence evolution of duplicated genes, and changes in DNA methylation. Such changes can result in novel phenotypes, ecological diversification, and new niche invasion (Otto and Whitton 2000). The acquisition of new functions is thought to facilitate novel reproductive systems, including conversion from dioecy to asexuality or selfing systems (Akagi *et al.* 2022). Nevertheless, the exact mechanisms that trigger transitions into and out of dioecy, via polyploidy, are not well understood.

## A hundred ways to derive chromosomal sex in plants

### Why so many pathways?

The discontinuity of dioecious plant lineages in evolutionary trees suggests that the determinants located in the sex chromosomes evolved independently (Ming *et al.* 2011, Renner 2014). Consistent with this assumption, physiological reactions to plant hormones, in terms of sex, can vary, with no clear commonality in direction. For instance, cytokinin (CK) treatment can enhance gynoecium growth in male grape accessions, resulting in the production of functional hermaphroditism (Negi and Olmo 1966, Wang *et al.* 2013). Similar sex conversion (or feminization) has been observed in crop species, including spinach (*Spinacia oleracea*) (Chailakhyan and Timiriazev 1979, Grant *et al.* 1994), hexaploid Oriental persimmon (*Diospyros kaki*) (Yonemori *et al.* 1993), kiwifruit (*Actinidia chinensis* or *deliciosa*) (Akagi *et al.* 2018), and wild plants, including the genus *Mercurialis* (Durand and Durand 1991), and *Plukenetia volubilis* (the family Euphorbiaceae) (Luo *et al.* 2020). Feminization can also be triggered via ethylene or auxin in hemp (*Cannabis sativa*), and via gibberellin in maize, as summarized by Grant *et al.* (1994). This situation is, at least partially, a result of differences in the reaction point in sex determination.

Various organs are potentially responsible for sex determination in plants (Dellaporta and Calderon-Urrea 1993). As represented by the two-mutation model (Charlesworth and Charlesworth 1978a), transitions into dioecy from ancestral functional hermaphroditism would require novel regulatory systems to sterilize either the gynoecium or androecium. Particularly for the androecium, there are various spatiotemporal procedures to form fertile gametes (Goldberg *et al.* 1993, Koltunow *et al.* 1990), from primordia formation in the shoot apical meristem to tapetum degradation during anther maturation. Any mutations in these many procedures could be a potential causal factor in the establishment of male or female, which might be a physiological reason that plants have recurrently evolved sexes in lineage-specific manners. For instance, androecium development in the female flowers of hemp is arrested in the primordia initiation stage (Mohan Ram and Nath 1964), while garden asparagus (*Asparagus officinalis*) or persimmon develop rudimentary anthers in female flowers, which fail during microsporogenesis (Akagi *et al.* 2014, Lazarte and Palser 1979). Kiwifruit and grapes exhibit “cryptically dioecious” systems (Mayer and Charlesworth 1991), in which female flowers look structurally hermaphroditic but are functionally unisexual, owing to pollen sterility that occurs during the pollen maturation stage (Akagi *et al.* 2019, Falasca *et al.* 2013, Lombardo *et al.* 1976, 1978). Despite these findings of morphological and physiological diversity in plant sex determination, the molecular pathways have only been characterized in a limited number of dioecious species. In this era of massive sequencing, there would be numerous results for a simple comparison of transcriptomic data between male and female. However, it is worth noting that physiological reactions in sex differentiation often differ among the organs or developmental stages, even under the regulation of a single sex determinant. In persimmon, an example of a well-characterized sex determination process, differentially expressed genes between males and females do not overlap in organ primordia initiation, organ development, and flower maturation stages (Li *et al.* 2019, Wang *et al.* 2020, Yang *et al.* 2019). Thus, with only physiological analyses, it is often hard to define a single molecular pathway for sex determination in a species.

### Sex determining genes and molecular pathways

Recent rapid progress in genomics has enabled the identification of sex chromosome-encoded determinants, which was previously challenging because of putative long non-recombining regions of sex chromosomes (Ming *et al.* 2011). The first finding of a sex chromosome-encoded determining gene in a plant was, interestingly, made in a wild persimmon, *Diospyros lotus* (or Caucasian persimmon) with no reference genome information (Akagi *et al.* 2014; Table 1). Cataloging of subsequences (or k-mer) in Illumina short reads, and a comparison of the male and female k-mer pools, resulted in direct isolation of Y chromosome-specific polymorphisms, including the determinants (Akagi *et al.* 2014). In persimmon (genus *Diospyros*), a Y chromosome-encoded small-RNA gene, named *OGI*, acts to suppress an autosomal counterpart gene, named *MeGI*, which is a HD-ZIP1 homeodomain gene. *MeGI* solely orchestrates both repression of androecium growth and promotion of gynoecium development, to integrate “feminization” (Yang *et al.* 2019). For androecium development, at least during primordia development, an *APETALA3* (*AP3*)/*PISTILLATA* (*PI*)-mediated pathway is repressed by *SHORT VEGETATIVE PHASE* (*SVP*)/*AGOUMOUS-like 24* (*AGL24*) and *AGAMOUS* (*AG*) (Gregis *et al.* 2009), putatively under the control of *MeGI* (Yang *et al.* 2019). *MeGI* can hypothetically enhance gynoecium development via direct regulation of the genes reminiscent of CK signaling, such as *KNOTTED-LIKE FROM ARABIDOPSIS THALIANA 1* (*KNAT1*), *OVATE FAMILY PROTEIN* (*OFP*). This was supported by the restoration of gynoecium in male flowers with CK treatment in hexaploid persimmon (Yonemori *et al.* 1993).

Garden asparagus (*Asparagus officinalis*) is a dioecious crop, in which sex is thought to be determined by two Y chromosome-encoded genes, *aspTDF1* (or *AoMYB35*) and *SUPPRESSOR OF FEMALE FUNCTION* (*SOFF*; with a domain of unknown function 247), acting as male-promoting (M) and female-suppressing (SuF) factors, respectively (Harkess *et al.* 2017, 2020, Murase *et al.* 2017, Tsugama *et al.* 2017). Whole genome sequencing of a doubled haploid 2A + YY individual, with the Illumina-PacBio-BioNano hybrid method, and resequencing of YY and XX siblings, found limited numbers of the sex determinant candidates in the hemizygous Y-specific region (Harkess *et al.* 2017). Further gamma irradiation-induced and EMS-induced mutants confirmed the two described genes as the sex-determining genes (Harkess *et al.* 2017, 2020). The gene *aspTDF1* is an ortholog of Arabidopsis R2R3-type Myb gene, *DEFECTIVE IN TAPETAL DEVELOPMENT AND FUNCTION1* (*TDF1*), which plays an important role in the degradation of the tapetal layer in anther maturation (Zhu *et al.* 2008). Consistent with its function in Arabidopsis, *aspTDF1* is substantially expressed in garden asparagus’ tapetal layer (Harkess *et al.* 2020), while its high expression has also been validated in early flower development (Tsugama *et al.* 2017). This situation is feasible because the female phenotype exhibits rudimentary anthers, which are derived from a lack of M factor functioning at the anther primordia stage. Nevertheless, molecular pathways regulated not only by *aspTDF* but also by *SOFF* are not well known.

Sexuality of kiwifruit (or the genus *Actinidia*) is also regulated by two genes in the Y-chromosome, named *Shy Girl* (*SyGI*) and *Friendly Boy* (*FrBy*), acting as SuF and M factors, respectively (Akagi *et al.* 2018, 2019). *SyGI* is a type-C *Arabidopsis Response Regulator 22/24* (*ARR22/24*)-like gene. *SyGI* negatively regulates the CK signaling pathway (Kiba *et al.* 2004) and affects the expression patterns of genes involving gynoecium identity or development, such as *AGAMOUS* (*AG*), *SHATTERPROOF* (*SHP*), *or SUPERMAN* (*SUP*) (Varkonyi-Gasic *et al.* 2021). *FrBy* is a gene with the fasciclin domain (FAS1 domain), which plays an important role in tapetum cell degradation for proper pollen maturation. The orthologs of *FrBy* are putatively monophyletic and functionally conserved in a range of angiosperm species (Akagi *et al.* 2019, Tan *et al.* 2012). *SyGI* arose recently via a lineage-specific duplication event that resulted in a new expression pattern in the gynoecium (Akagi *et al.* 2018). This evolutionary pattern is thought to be similar to that in garden asparagus, which is consistent with the scenario proposed in the two-mutation model (Charlesworth and Charlesworth 1978a). The timeline for establishment of the two factors, which could be important for defining their evolutionary significance, remains unknown. The closest outgroup lineage (genus *Saurauia*, in the family Actinidiaceae) is also dioecious (Haber and Bawa 1984), and the sex determinant would be independent of the genus *Actinidia* (Akagi *et al.* 2018).

Poplar (*Populus* species) has multiple dioecious mechanisms, which grouped into both XY (heterogametic male) and ZW (heterogametic female) systems in lineage-specific manners during evolution of the genus (Müller *et al.* 2020). Translocations, duplications, or simple mutations of a single ancestral regulator, *ARR17*, have formed various structures to cause unisexuality at least three times in the genus *Populus* (Müller *et al.* 2020). However, other possibilities cannot be excluded, such as a potential two-gene model in *P. deltoides* (Xue *et al.* 2020). ARR17 is a type-A *Arabidopsis Response Regulator* (*ARR*), with functions that may be overlapped with *SyGI* (type-C *ARR*) in terms of negative regulation of CK signaling. However, their structures are quite distant (Kiba *et al.* 2004, To *et al.* 2004, To and Kieber 2008). CRISPR-Cas9-mediated gene-editing of rapid flowering *P. tremula*, revealed that *ARR17* appears to repress *UNUSUAL FLORAL ORGANS* (*UFO*) expression, which activates B-class MADS-box genes, including *PISTILLATA* (*PI*); these genes are required for androecium development (Leite Montalvão *et al.* 2022). The expression networks in *Populus* are quite distinct from those in kiwifruit (Varkonyi-Gasic *et al.* 2021), the differences lying in the reaction points of the sex determinants.

Recent studies identified sex determinant candidates in some other crops, including grapevine (*Vitis* species) and date palm (*Phoenix* species). Cultivated grapevine species (*V. vinifera*, *V. labrusca*, and their hybrid *V. labruscana*) are hermaphroditic (as discussed in detail later), while wild grapes are mostly dioecious. Efforts to trace the history of recombination from which hermaphroditism was derived in cultivated grapevines, suggest limited numbers (fewer than 10) of two-factor sex determinant candidates in a small sex-linked region (>150 kb) (Badouin *et al.* 2020, Massonnet *et al.* 2020, Zhou *et al.* 2017). Importantly, no candidates in the grapevine overlap with the sex determinants identified in other species. However, Badouin *et al.* (2020) suggested that regulation of gynoecium development (or function of SuF) might involve CK signaling. A comparative genome analysis of dioecious date palm species and monoecious oil palm revealed the sex determination region in the Y chromosome, which included the three most likely candidates (Torres *et al.* 2018). Of these, a duplicated *LONELYGUY* (*LOG*)-like gene, which putatively involves CK activation (Kuroha *et al.* 2009), is thought to be an SuF candidate. These situations may indicate a potential link among dioecious species for the function of gynoecium regulators, i.e., they commonly affect CK metabolism or signaling.

### Theoretical models and sex chromosome evolution

No uniform evolution has been proposed for plant sex determination, either in terms of molecular mechanisms or theoretical frameworks. The “two-mutation” theory (Charlesworth and Charlesworth 1978a, Westergaard 1958) is a representative model that explains many dioecious systems, and has been validated in garden asparagus (Harkess *et al.* 2017) and kiwifruit (Akagi *et al.* 2019). However, sex determination in persimmon, in which the Y-encoded smRNA *OGI* acts alone, is apparently distinct from the canonical two-gene model. This single-gene framework could be a result of the bidirectional function of the target of *OGI*, *MeGI*, which simultaneously represses and promotes androecium and gynoecium development, respectively (Yang *et al.* 2019). From an evolutionary aspect, this *OGI–MeGI* interactive system may share a consistent concept with the two-mutation model (Charlesworth 2019). A single-gene system is also applicable to *P. tremula*, in which gene-editing of the Y-encoded *ARR17* resulted in conversion from male to female (Müller *et al.* 2020). In contrast with the two-mutation model, concrete evolutionary paths for the single-gene system have not yet been defined. An assumption for this evolution is the conversion from monoecy to dioecy, by establishment of a genetic factor to complement a biased female/male ratio in an individual (Charlesworth 2013), which would be consistent with the artificial evolution of dioecy in Cucurbit (Boualem *et al.* 2015).

Recent progress in DNA sequencing techniques has enabled chromosome-scale draft genome construction, even in dioecious species with sex chromosomes (e.g., Harkess *et al.* 2017, 2020 for asparagus, Akagi *et al.* 2019, 2023 for kiwifruit, Akagi *et al.* 2020 for persimmon, Ma *et al.* 2022 for spinach). With these genomic results (along with functional analyses of sex determining genes), evolutionary mechanisms to form sex chromosomes in plants may have to be reconsidered. Conventional theory suggests that gradual expansion of a non-recombining region or structural degeneration of the Y chromosome would be a result of maintenance of linkages between the sex determining gene and the surrounding sexually antagonistic genes, or maintenance of sexual dimorphism. Thus, the age of sex chromosomes is thought to be important for their degree of heteromorphism. This hypothesis is, at least partially, supported by some dioecious species (Ming *et al.* 2011). However, the recent accumulation of sequencing data of sex chromosomes suggests that this tendency might not be common in plant species (Renner and Müller 2021). Furthermore, functional analyses of sex-determining genes from persimmon and kiwifruit suggest that representative sexual dimorphisms in these species can be explained by the pleiotropic functions of sex determining genes, and be independent of genes surrounding the sex determinants (Akagi and Charlesworth 2019, Akagi *et al.* 2023). Importantly, a recent theoretical model also proposed the potential independence of Y chromosome degeneration and selection of sexual dimorphisms (Lenormand and Roze 2022). There are clearly still many mysteries in plant sex chromosome evolution.

## What makes a crop a crop? Transitions out of dioecy

### Impact of domestication

Domestication is the most important process of selection by humans which transformed wild plant forms into crops. The gradual transition from hunting and gathering to plant cultivation and animal husbandry began between the end of the Pleistocene and the beginning of the Holocene, some 12,000–10,000 years ago (Arranz-Otaegui *et al.* 2018, Fuller *et al.* 2014). As a result of this process, crop plants and domesticated animals share suites of modified traits, referred to as ‘domestication syndrome’, which differentiates them from their wild ancestors (Meyer *et al.* 2012, Stetter *et al.* 2017). In plants, domestication syndrome often involves changes in plant architecture, shattering, the sugar content of fruit, fruit size, and the shift from outcrossing sexual systems. Of these changes, the shift from an outcrossing system is thought to be a requirement for the stable production (cultivation) of a crop with extremely low genetic diversity, as first implied by Darwin (1877b). Although a number of agronomically important plants still exhibit dioecy (see Table 1) (and other outcrossing systems, such as self-incompatibility or dichogamy), some major horticultural crop species successfully switched from dioecious progenitors to self-fertile hermaphroditism during domestication, e.g., grape, papaya, carob, and strawberry. This switch was likely driven by selection for reproductive assurance (Crossman and Charlesworth 2014).

### Artificial selection of hermaphroditism in domestication

Wild populations of papaya are strictly dioecious, while cultivated populations are almost always gynodioecious, including hermaphroditic and female individuals (Brown *et al.* 2012). These findings imply a breakdown of dioecy during domestication. Hermaphroditic papaya plants reliably produce fruit because they can self-pollinate and do not require male plants. In addition, the shape of hermaphroditic fruit, which is more elongated than female fruit, is preferred for commercial production (Liao *et al.* 2021). Sex determination in papaya is controlled by a recently evolved heterogametic male system (or XY system). A putatively mutated Y chromosome, named Y^h^, corresponds to hermaphroditic individuals (Liu *et al.* 2004, Ma *et al.* 2004, Wang *et al.* 2012). All genotypes without an X chromosome (YY, YY^h^, and Y^h^Y^h^) die in early development, resulting in 25% aborted seeds in self-pollination of hermaphrodites and in crosses between hermaphrodites and males (Ming *et al.* 2007). This finding indicates that both normal and mutated Y alleles have lost at least one gene essential for development. The Y^h^ chromosome has a non-recombining male-specific region (MSY), as does the normal Y chromosome, which limits the hermaphroditic-specific region of Y^h^ (HSY) (VanBuren *et al.* 2015, Wang *et al.* 2012). The Y and Y^h^ chromosomes share much of their genomic context (99.6% sequence similarity), except in the hermaphrodite-specific HSY region. Y^h^ was putatively derived from mutation of the female suppressing factor (SuF), as proposed in the two-mutation model. Importantly, the HSY region including the mutated SuF, exhibits low genetic diversity and some indexes for selective sweeps. These findings suggest artificial selection of hermaphroditism during the domestication process in Costa Rica, approximately 4000 years ago (VanBuren *et al.* 2015). Some studies have proposed positive disruption of dioecy during domestication, while population genetic research on papaya HSY provides direct evidence for historical artificial selection of a breakdown of dioecy to functional hermaphroditism.

The genus *Vitis* includes approximately sixty species in the subgenera *Muscadinia* and *Euvitis*, all of which are dioecious except for cultivated grapes. During grapevine domestication, flower reproductive morphology has been modified, with the transition from dioecy to hermaphroditism (This *et al.* 2006). Similar to papaya, hermaphroditism in cultivated grapevine is also thought to be a result of a mutated allele of the Y chromosome, named the H allele. The H allele appears to lack the SuF gene and is derived from a part of the Y chromosome in wild male grapevines (Picq *et al.* 2014). Population genetics approaches have identified indexes of selective sweeps on loci that appear to be associated with hermaphroditism, as well as berry color, implying artificial selection during domestication (Zhou *et al.* 2019). Genome sequencing analysis across 556 genotypes found two distinct hermaphrodite haplotypes (H1 and H2) among the cultivated grapevines, both derived from chimeras of male (M) and female (f) haplotypes (Zou *et al.* 2021). H1 haplotypes are distributed globally and are common in table, raisin, and wine grapes. H2 haplotype is only found in a group of wine grape cultivars in a limited area (north and west Europe). The time of divergence between the H1 and H2 haplotypes is estimated to be ~6 million years ago (Zou *et al.* 2021), which apparently predates the domestication of grapevines (~8,000 years ago). This inconsistency might suggest two independent selection histories of these hermaphrodite haplotypes.

### Escape from a dioecious system associated with polyploidization

The genus *Diospyros* consists of about 400 species that are widely distributed in the (sub)tropical areas of Asia, Africa, and the Americas, and are generally dioecious. An exception to this dioecy is a cultivated hexaploid species, *D. kaki* (2n = 6x = 90), known as Oriental persimmon, which is considered to be polygamous, with three types of sex expression: gynoecious, monoecious, and polygamo-monoecious (hermaphroditic, female, and male flowers in an individual). In *D. kaki*, genetically female individuals (or individuals with hexaplex X: 6A + 6X) produce no male flowers, as well as in diploid wild relatives. Genetically male individuals (or individuals carrying at least one Y chromosome) can exhibit monoecious expression (Akagi *et al.* 2016b). In the Y chromosome of *D. kaki*, the sex determinant *OGI* was fundamentally silenced by the insertion of a short interspersed nuclear element (SINE)-like TE, named *Kali*, into its promoter region (Akagi *et al.* 2016a). Instead, the autosomal counterpart of *OGI*, *MeGI*, established a novel expression regulatory system based on DNA methylation in its promoter region that acts as an epigenetic switch to produce male or female flowers (Akagi *et al.* 2016a). Importantly, the *Kali* SINE in the *OGI* promoter is conserved in all accessions with a Y chromosome, including landraces that hypothetically have not experienced domestication events and exhibit no clear signals for recent selection. This situation would suggest that the conversion from male to monoecy was independent of domestication and might be coincident with (or possibly predate) the hexaploidization event.

**Fig. 1.**
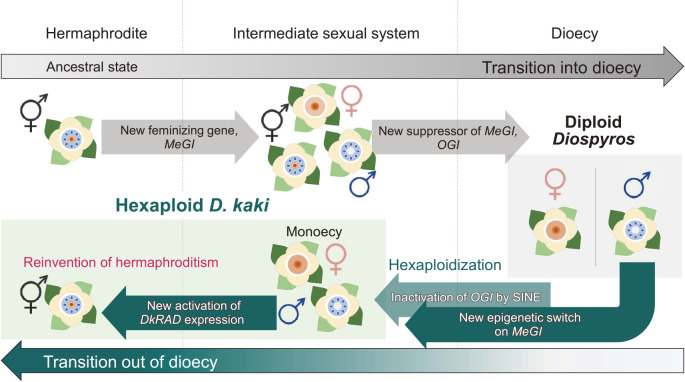
Scrap and (re)build of sex systems in the genus *Diospyros*. Dioecy in *Diospyros* species putatively evolved from ancestral hermaphroditism. Transitions into intermediate sexual systems (possibly monoecy) and into dioecy were triggered by acquisition of two neofunctionalized genes: *MeGI*, a new feminizing gene, and *OGI*, a suppressor of *MeGI*. Hexaploid *D. kaki* evolved a monoecious phenotype from genetic males, via inactivation of *OGI* by a SINE insertion and acquisition of a new epigenetic switch on *MeGI*. Additionally, new activation of *DkRAD* expression triggered reversions from male to hermaphroditic flowers, which is perfectly independent of the existing *OGI-MeGI* system.

The hexaploid *D. kaki* has further invented a new pathway to occasionally convert male flowers to hermaphroditic ones, independent of the *OGI*–*MeGI* system (Masuda *et al.* 2022). This sex conversion can occur naturally or be induced by cytokinin treatment (Yonemori *et al.* 1993). The mechanism depends on hexaploid-specific activation of the cytokinin- or abscisic acid-responsive signaling pathways and their putative integrator, *RADIALIS*-like *DkRAD* (Masuda *et al.* 2022). Notably, this *DkRAD*-mediated hermaphrodite expression occurs randomly in a tree and has not been utilized for fruit production, suggesting no involvement in the domestication/improvement process. The *RADIALIS* gene is well known as a regulator in flower morphology, particularly petal architecture (Lucibelli *et al.* 2020). Hence, the *DkRAD* function in persimmon as a sex converter could be a result of a neofunctionalization (Bergero 2022). Although the expression of hermaphroditism in *D. kaki* may look like a reversion to the ancestral state via loss of the existing pathway, this evolution could be a reinvention of hermaphroditism via establishment of a novel pathway. As shown in Fig. 1, persimmon provides a good example of frequent “scrap & rebuild” evolution of sex expression, which may be triggered by ancient and recent duplication events (Masuda *et al.* 2022).

## Perspective: the power of duplication

We focus on gene or genome-wide duplication as a potentially common mechanism to trigger transitions into and out of dioecy in plants. The involvement of genome-duplication (or polyploidization) in such transitions has been theoretically discussed, based on available literature on sexual systems in various plant species (Glick *et al.* 2016, Goldberg *et al.* 2017). Recent genome duplications or polyploidization events have also enabled plasticity in plant sexual reproduction systems, including sexuality (Ashman *et al.* 2013, Comai 2005). The focus on duplications is due to the nature of plant genome behavior. Plants have undergone frequent genome-wide duplications, in a lineage-specific manner, as exemplified by the frequent paleo-ploidizations in the Cretaceous–Paleogene (K-Pg) boundary (Van de Peer *et al.* 2017). Genome duplications rapidly produce various phenotypic consequences associated with gene expression variation—resulting from novel allele dosages or regulatory interactions—or with genetic and epigenetic rearrangements/modifications (Fox *et al.* 2020, Osborn *et al.* 2003, Van de Peer *et al.* 2021, Wendel 2000). In the long term, duplicated gene pairs often actively exhibit sub-functionalization, pseudogenization, or neofunctionalization, mainly to release them from functional redundancy or adaptive conflicts (Des Marais and Rausher 2008, Flagel and Wendel 2009, Ohno 1970). In other words, a lineage-specific duplication can provide an opportunity to acquire novel functions or traits representing each species. For instance, oil production in olive (*Olea europaea*) (Unver *et al.* 2017), fruit ripening processes in tomato (*Solanum lycopersicum*) (Tomato Genome Consortium 2012), or adaptation to the sea in seagrass (*Zostera marina*) (Olsen *et al.* 2016), were reportedly established via genome duplications specific to each lineage, followed by dynamic functional diversifications between the duplicated genes.

**Fig. 2.**
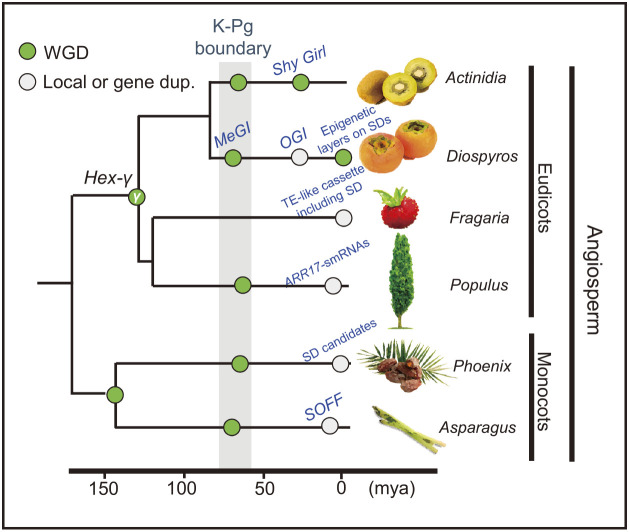
Lineage-specific duplications involving new sexual systems or sex determinants. Whole genome-wide duplications (WGD, light green circles) and local genome or gene duplications (white circles) are shown in the schematic phylogenetic tree. The ages (mya: millions of years ago) of each WGD are adapted from Van de Peer *et al.* (2009), Huang *et al.* (2013), Vanneste *et al.* (2014), Harkess *et al.* (2017), Van de Peer *et al.* (2017), and Akagi *et al.* (2020). The lineage-specific duplications that produced sex determinants (SD) or events involving new sexual systems, are annotated with their functions or gene names in blue (Akagi *et al.* 2014, 2016a, 2018, 2019, Harkess *et al.* 2017, 2020, Müller *et al.* 2020, Tennessen *et al.* 2018, Torres *et al.* 2018).

The evolutionary processes involved in the establishment of sex determinants could also be explained by genome or gene duplications to invent novel functions (summarized in Fig. 2). As partially suggested in the previous sections, in persimmon, the Y-encoded *OGI* was derived from gene duplications to form an inverted repeat (or small-RNA encoding locus), which is specific to the genus *Diospyros* (Akagi *et al.* 2014). The target of *OGI*, *MeGI*, was neofunctionalized to act as a feminization factor via a lineage-specific genome-wide duplication (or paleo-polyploidization) at the K-Pg boundary (Akagi *et al.* 2020). The genus *Asparagus* underwent multiple genome-wide or partial genome duplication. A recent duplication that is specific to dioecious lineages, such as garden asparagus, derived the Y-encoded *SOFF* gene (Harkess *et al.* 2017). *Shy Girl* in the genus *Actinidia* also arose from an *Actinidia*-specific genome-wide duplication (Huang *et al.* 2013), and resulted in novel expression patterns in the gynoecium (Akagi *et al.* 2018, 2019). Furthermore, in the family Salicaceae, multiple recent lineage-specific duplications of *ARR17* formed inverted repeats (or small-RNA coding loci), resulting in at least three independent Y-encoded sex-determining loci (Müller *et al.* 2020). Extending this concept, frequent duplications (or translocations) of a transposable cassette putatively including the sex determinants in the genus *Fragaria*, triggered various lineage-specific sex determination systems, with the formation of neo-sex chromosomes (Tennessen *et al.* 2018). There are two potential mechanisms through which these duplications could trigger the establishment of sex determinants: (i) a novel SuF (in the “two-mutation” model) or a dominant suppressor, which is often essential for the establishment of dioecy, is thought to be hard to generate from a single (non-redundant) gene; and/or (ii) transition to dioecy may need dynamic changes in the regulatory path adjusted/specialized to each sex, represented by sexual dimorphisms, to which genome-wide duplication(s) may substantially contribute.

Regarding the first hypothesis, transitions other than those in the two-mutation model (e.g., from monoecy to dioecy) might not necessarily involve dominant suppressors but rather work only with a simple loss of function, as indicated by artificial evolution in Cucurbit (Boualem *et al.* 2015). Most of the identified paths to XY system dioecy include at least one dominant suppressor, as described, but the mechanisms remain unsolved. With the exception of genes that are highly redundant for their function (although these would often be generated by recent duplications), mutations that alter a single gene to act in a new distinct function (such as a dominant suppressor) would be evolutionarily disadvantageous, owing to the loss of the original function(s) (Flagel and Wendel 2009, Wendel 2000). However, duplication also provides a chance to establish functional redundancy and facilitate neofunctionalization into a dominant suppressor. Consistent with this hypothesis, a genome-wide duplication in the ancient lineage of the genus *Diospyros* (or the family Ebenaceae), named *Dd*-α, derived a paralogous pair of HD-ZIP1 type homeobox genes. One of these duplicates underwent strong positive selection on specific mutated residues to be neofunctionalized into the current *MeGI*, which is a novel dominant suppressor of male function (Akagi *et al.* 2020). In terms of the second hypothesis, there are no clear genetic or physiological clues yet. In whole-genome duplication (or even simple gene duplication) events, the evolution of *cis*-regulatory elements has made more rapid and substantial contributions to lineage-specific acquisition of representative traits than that of *trans*-acting elements in plants (Charoensawan *et al.* 2010, Roulin *et al.* 2013), as well as in animals (Carroll 2008, Lynch and Conery 2000, Wray *et al.* 2003). In actuality, numerous paralogous gene pairs exhibit fundamental differentiation in the expression patterns in flower buds, represented by the *Dd*-α in persimmon (Akagi *et al.* 2020). This finding is in contrast to limited gene pairs with strong positive selection potentially deriving neofunctionalized *trans*-functions. However, the functions of these differentially expressed genes, particularly involving sex expression, are not well defined. In the future, if a new determinant involving the establishment of dioecy (or even escape from dioecy) were to be derived via genome duplication, we may have to focus not only on the sex determining genes but also on a birds-eye view of “the whole” genome evolution, such as genome/epigenome rearrangements adjusted to the transitions into new sexual systems. Genome-wide surveys for changes in evolutionary pressures (or evolutionary rates: *dN/dS*) or expression patterns between ancestral and new sexual systems could also provide insights into the adaptations to new sexual systems. These would accelerate our understanding of the evolution of sex determination systems in plants, as well as providing future directions to harness various plant sexualities for breeding, via recent genome techniques, such as gene-editing.

## Author Contribution Statement

K.M. and T.A. wrote the manuscript.

## Figures and Tables

**Table 1.** Sex determination systems in representative dioecious crops

Taxonomy-Order	Genus	Sex detemination system	Sex determination model	Sex detemining genes	Major crop	Sexual systems in crop	references
Eudicots-Ericales	*Diospyros*	XY	single factor	*OGI*	Oriental persimmon (*D. kaki*)	Female and monoecious individuals. Male flowers are often converted to hermaphrodite flowers.	Akagi *et al.* (2014, 2016a) Masuda *et al.* (2022)
Eudicots-Ericales	*Actinidia*	XY	two factors	*Shy Girl* *Friendly Boy*	Kiwifruit (*A. chinensis* or *deliciosa*)	Dioecious	Akagi *et al.* (2018, 2019)
Eudicots-Malpighiales	*Poplus*	almost XY (*P. tremula* etc.)	single factor	*ARR17* inverted repeat	Poplar (*P. trichocarpa*)	Dioecious	Muller *et al.* (2020)
		XY (*P. deltoides* etc.)	two factors	*FERR-R* *MSL*	—		Xue *et al.* (2020)
		ZW (*P. alba* etc.)	single factor	*ARR17*	—		
Eudicots-Rosales	*Fragaria*	ZW (*F. virginiana*, *F. chiolensis* etc.)	Tranposable sex determination cassette	*GMEW* (candidate)	Strawberry (*F. × annanasa*)	Cultivated species are hermaphrodite.	Liston *et al.* (2014) Tennessen *et al.* (2018)
Eudicots-Rosales	*Cannabis*	XY	X-A balance	unknown	Hemp (*C. sativa*)	Monoecious and dioecious cultivars.	Faux *et al.* (2014)
Eudicots-Rosales	*Humulus*	XY	X-A balance	unknown	Hop (*H. lupulus*)	The sex determination mechanism is shared with the genus *Cannabis*.	Prentout *et al.* (2021)
Eudicots-Brassicales	*Carica*	XY	two factors	unknown	Papaya (*C. papaya*)	Major cultivars are hermaphrodites.	Wang *et al.* (2012) Van Buren *et al.* (2015)
Eudicots-Vitales	*Vitis*	XY	two factors	*VviYABBY3*, *VviINP1* (candidates)	Grape (*V. vinifera* or *labruscana*)	Cultivated species are mainly hermaphrodite.	Massonnet *et al.* (2020)
Eudicots-Caryophyllales	*Spinacia*	XY	single factor	*NRT1* (candidate)	Spinach (*S. oleracea*)	Dioecious	Ma *et al.* (2022)
Eudicots-Caryophyllales	*Silene*	XY (*S. latifolia*)	two factors	unknown			
		ZW (*S. otites*)		unknown			
Eudicots-Caryophyllales	*Rumex*	XO	X-A balance	unknown			
Eudicots-Sapindales	*Pistacia*	ZW		unknown	Pistachio (*P. vera*)	Dioecious	Kafkas *et al.* (2015)
Monocots-Asparagales	*Asparagus*	XY	two factors	*SOFF* *TDF1*	Garden asparagus (*A. officinalis*)	Dioecious	Harkess *et al.* (2017, 2020)
Monocots-Arecales	*Phoenix*	XY	two factors	*LOG* *CYP703*, *GPAT3* (candidates)	Date palm (*P. dactylifera*)	Dioecious	Torres *et al.* (2018, 2021)
Monocots-Dioscoreales	*Dioscorea*	almost XY (*D. alata*, *D. tokoro* etc.)		unknown	Water yam (*D. alata*)	Dioecious	Sugihara *et al.* (2021)
		ZW (*D. rotundata*, *D. deltoidea*)		unknown	Guinea yam (*D. rotundata*)	Dioecious	
		XO (*D. sinuata* etc.)		unknown	—		
